# Sensors for Lung Cancer Diagnosis

**DOI:** 10.3390/jcm8020235

**Published:** 2019-02-11

**Authors:** Rosamaria Capuano, Alexandro Catini, Roberto Paolesse, Corrado Di Natale

**Affiliations:** 1 Department of Electronic Engineering, University of Rome Tor Vergata, 00133 Roma, Italy; capuano@ing.uniroma2.it (R.C.); catini@ing.uniroma2.it (A.C.); 2 Department of Chemical Science and Technology, University of Rome Tor Vergata, 00133 Roma, Italy; roberto.paolesse@uniroma2.it

**Keywords:** lung cancer, biosensors, volatile compounds, electronic nose

## Abstract

The positive outcome of lung cancer treatment is strongly related to the earliness of the diagnosis. Thus, there is a strong requirement for technologies that could provide an early detection of cancer. The concept of early diagnosis is immediately extended to large population screening, and then, it is strongly related to non-invasiveness and low cost. Sensor technology takes advantage of the microelectronics revolution, and then, it promises to develop devices sufficiently sensitive to detect lung cancer biomarkers. A number of biosensors for the detection of cancer-related proteins have been demonstrated in recent years. At the same time, the interest is growing towards the analysis of volatile metabolites that could be measured directly from the breath. In this paper, a review of the state-of-the-art of biosensors and volatile compound sensors is presented.

## 1. Introduction

Lung cancer is one of the most lethal cancer forms, in particular because a large part of the development of the disease may occur in an asymptomatic form, and the disease is typically manifested at advanced stages [1]. Thus, early detection is a key issue in lung cancer. Low-dose chest computed tomography (CT) scanning has been suggested as a screening tool, especially in the presence of high risk factors for lung cancer [2]. This type of procedure, carried out every year, is expected to increase greatly the possibility of early-stage tumor diagnosis, contributing to the increase of the survival rate [3]. However, the technique is affected by a large rate of false positives [4]. Furthermore, the exposure to the ionizing radiation of low-dose CT might increase the risk of developing cancers. Thus, large population screening, even if limited to people considered at risk, requires an alternative solution that should be non-invasive and low cost. Furthermore, the simplicity of use should also be considered to ensure that a large strata of health operators can adequately utilize the instrumentation.

The technology of sensors combined with microelectronics enables the miniaturization of complex instruments in many different fields. The gradual development of sensors for diagnosis is devised as one of the major outcomes of technology in medicine.

In the case of lung cancer, sensor development is mainly oriented towards two different scopes: the detection of specific cancer-related biomarkers and the detection of patterns of volatile metabolites.

The two approaches, although aimed at detecting molecules linked to cancer, are qualitatively very different. Indeed, the first is the case of specific sensors for the detection of well-defined molecular targets. In the second case, the object of the measurement is a pattern of volatile compounds, and for this task, arrays of sensors are used.

In this paper, the sensor technologies for both of these objectives are introduced, and the state-of-the-art of sensors for the identification of lung cancer is presented and discussed.

## 2. Introduction to Sensors

Electronic sensors are devices that convert the information about a generic quantity into an electric signal. Thus, a chemical sensor produces an electric signal (typically a voltage) that is a function of the concentration of the chemical compounds with which the sensor interacts. For this scope, chemical sensors are made of two components: a chemically-interactive material and a basic sensor device. The chemically-interactive material is a layer where the interaction with the target molecules takes place, while the basic sensor device is the actual sensor that measures a physical quantity that is affected by the interaction [5].

In chemical sensors, the chemically-interactive material is typically either a polymer or a molecular film. The basic sensors may detect changes of mass, electric conductivity, or optical properties (absorbance or fluorescence).

An important methodology in chemical sensors, particularly advantageous to measure complex samples characterized by a large number of compounds, is represented by the arrays of cross-selective sensors. These systems are directly inspired by the sense of olfaction. Indeed, in nature, the diversity of molecules for which the olfaction has to be sensitive is extremely large, and they are always combined together. Namely, odors are not made of a single chemical species, but of a blend of different species. Nature solved the problem of detecting this myriad of different combinations by using an ensemble of non-selective sensors. Specifically, a single olfactory receptor is sensitive to more molecules, but olfactory receptors are different from each other [6]. In this way, the collective olfactory receptors encode odors into patterns of signals that are specific for each odor [7]. This strategy is called combinatorial selectivity, and it can be adequately reproduced with artificial sensor arrays, also known as electronic noses.

The special cases when the chemically-interactive material is made of biochemical molecules are called biosensors. Enzymatic sensors and immunosensors are the two main categories of biosensors.

In the first case, the product of catalytic reactions promoted by an enzyme are measured, while immunosensors exploit the ligand properties of proteins, antibodies, and DNA strands. Enzymatic sensors include the biosensors for the measurement of glucose in blood, while in the second case, sensors are designed to reproduce, at a smaller scale, immunoenzymatic tests such as the enzymatic immunosorbent assay (ELISA).

The performance of sensors is measured by parameters such as the sensitivity and the limit of detection [8]. The sensitivity is defined as the rate of variation of the sensor signal with respect to the variation of the analyte, and the limit of detection expresses the smallest concentration of the analyte that elicits a readable signal.

Electronic noses are used to classify samples into groups such as: lung cancer affects subjects and control individuals. For this scope, it is necessary to use a proper machine learning algorithm. These are the same techniques used in metabolomics studies [9]. It is beyond the scope of this paper to review the machine learning methods; however, it is clear that classification algorithms are based on accumulated experience, and then, it is absolutely necessary to validate the data analysis models with independent datasets [10].

The performance of electronic noses is reported through quantities that measure the correct identification of samples as belonging to a specified group. The most used quantities are the true positive rate (TPR), the true negative rate (TNR), and the accuracy. In medical studies, it is well established that TPR and TNR are also called the sensitivity and specificity. These terms have to be carefully used in order to avoid confusion with the nomenclature in use in analytical sciences. They are defined as follows:

$$Sensitivity:\;\frac{TP}{TP+FN};\;Specificity=\frac{TN}{TN+FP};Accuracy=\frac{TP+TN}{P+N}$$

where *P* and *N* are the total positive and negative cases; *TP* (true positive) and *TN* (true negative) are those correctly recognized as positive or negative, respectively; *FP* (false positive) and *FN* (false negative) are the positive and negative cases that are wrongly identified.

## 3. Biosensors for Biomarkers

In the past few years, a number of proteins whose expression is related to different forms of cancers have been identified [11]. The development of biosensors requires the immobilization of a layer of recognition elements, typically antigens, antibodies, or DNA strands, on the inorganic sensor surface. The immobilization procedure is not simple, in particular because the tridimensional shape of the receptor is usually lost when it is immobilized on a surface. The denaturation of the receptor leads to the loss of the molecular recognition property. On the other hand, even when the immobilization is successful, due to the complexity and fragility of biomolecules outside their natural environment, biosensors often suffer from instability and a short lifetime. Nonetheless, the research on biosensors is strongly progressing, and the positive examples, still at the research level, are expected to end up in routine products [12].

Among the markers for lung cancer, it is worth mentioning the cytokeratin fragment 21-1 (CYFRA21-1) and the neuron-specific enolase (NSE). These two markers are known to differentiate between the two major forms of lung cancer: non-small cell and small cell lung cancers [13]. These molecules can be identified in serum with standard immunosorbent assays. However, these methods require labelling the target molecule in order to be identified.

Biosensors can be developed for a fast label-free detection of the immunoselective interaction. Cheng et al. demonstrated a field effect transistor-based biosensor where the human antigen CFYFRA21-1 and NSE are bonded on their sensitive surface, a silicon transistor whose size is 10 µm × 1000 µm [14]. The minimal quantity required, the lack of labelling, and the high sensitivity of the device enable the detection of these markers at concentrations of about 1 ng/mL.

More recently, a different solution based on the color change of gold nanoparticles coated with antigens has been introduced [15]. The limit of detection of this sensor was less than 1 ng/mL. This last method was also demonstrated to be efficient for the label-free detection of Dickkopf-1, whose putative relationship with lung cancer has been suggested [16].

Another important target for biosensor detection is the “epidermal growth factor receptor” (EGFR); the detection of the mutation of this protein provides valuable information for the detection and the management of non-small cell lung cancer [17]. The detection of ThFR mutations has been demonstrated with an integrated optical device incorporating the DNA sequence related to genetic mutation [18]. The overall detection, including the DNA amplification, is characterized by a limit of detection of 0.125 pg/µL, at least one order of magnitude smaller than the conventional analysis.

It is interesting to consider that biomarkers could also be detected in different fluids. Exhaled breath condensate has attracted attention due to the minor invasiveness of the sampling [19].

To this regard, a surface acoustic wave-based immunosensor was developed to detect carcinoembryonic antigen (CEA) in exhaled breath condensate with a limit of detection below 1 ng/mL [20]. CEA is a glycoprotein known to play a role in colon carcinoma metastatic spread and also found to be predictive of lung cancer [21].

## 4. Analysis of Volatile Metabolites in Breath

The relationship between volatile compounds in breath and lung cancer has been shown by a number of different studies [22]. However, the search for molecular markers related to specific diseases has resulted in poor evidence. Indeed, except for some particular conditions (e.g., acetone for diabetes), the pathological state is signaled rather than a single molecule by a pattern of volatile compounds [23,24].

A critical aspect of breath analysis is the collection of the sample. Obviously, the sample can be highly contaminated from food, smoke, and in general, environmental air. Thus, even if the measurement is absolutely non-invasive, it requires a non-negligible contribution of the subject to follow some rules about food and hygienic procedures. It is important to note that this aspect is still non-standardized, and then, the comparison between different experiments should also include the different measurement conditions [25]. In the case of lung cancer, it is crucial to analyze only the alveolar portion of the breath. An accurate separation of alveolar breath from the so-called dead space can be achieved measuring, in real time, the content of CO_2_ [26]. Since this requires the use of an additional sensor, in practical cases, alveolar breath separation can be simply obtained by discarding the first fixed volume of breath [27].

The chemical composition of breath is largely influenced by environmental chemistry. Inhaled air contains, besides oxygen and nitrogen, all the compounds that characterize the environment. On the contrary, exhaled air is characterized by a small concentration of oxygen, a large content of carbon dioxide, and a number of volatile compounds produced by the body metabolism.

The abnormal metabolism of tumor cells is expected to alter not only the quantity, but also the quality of the excreted metabolites. The relationship between the composition of exhaled breath and lung cancer has been investigated for three decades. Gas chromatography has been the gold standard in these studies [28,29,30,31]. However, the results of such measurements depend on a series of factors that include the choice of the absorbent material, the setup of the gas chromatograph, and the characteristics of the detector, among others. In practice, since the measure of breath is not standardized, each investigation captures different aspects of the breath composition, and a clear identification of the involved volatile compounds is still missing [32]. A review of lung cancer-related volatile organic compounds (VOCs) enumerate 19 compounds whose concentration has been found to be statistically altered in the presence of cancer. These compounds belong to a large spectrum of chemical diversity [22].

In spite of the growing collection of evidence, the relationship between cancer and VOCs is still debated. The main hypothesized pathways for the formation of cancer-related VOCs include oxidative stress, gene mutations, and the Warburg effect [33].

Oxidative stress is the result of the increased activity of the cytochrome P450 enzyme [34]. This leads to an excess of concentration of reactive oxygen species and free radicals. These species can react with different molecules in the body, giving rise to a number of compounds that can be emitted in the breath [35]. On the other hand, oxidative stress is common to many inflammatory stages, and so, it cannot be considered a specific source of VOCs for cancer.

Gene mutations are typically associated with the growth of cancer. The mutations result in altered proteins that may induce changes in the metabolic processes and ultimately result in abnormal production of VOCs [36]. Another supposed source of VOCs is the Warburg effect, which corresponds to the glycolysis over oxidative phosphorylation that is activated in hypoxic condition [37]. The reduction of available oxygen is likely a consequence of fast cell replication [38]. This reaction produces an excess of lactic acid that may causes the breakage of the basement membrane, which eventually results in the production of small detectable volatile molecules [39].

On the other hand, all these studies agree in considering that the pattern is altered and that there is not a clear relationship between the molecular structure and the relevance for cancer. For instance, the concentration of 1-methyl-2-(1-methylethyl)-benzene does not depend on cancer, while the abundance of 1-methyl-3-(1-methylethyl)-benzene is five-times larger in the breath of cancer-affected individuals [31]. In spite of the difficulties in finding a relationship between the chemistry of volatile compounds and cancer, the identification of a pattern signaling cancer can be achieved by electronic noses.

Several gas sensors technologies have been applied to breath analysis [40]. In the case of lung cancer, the first evidence that an electronic nose can identify cancer from the analysis of breath was obtained in 2003 using an array of porphyrin-coated quartz microbalances [41]. Porphyrins are known for the important role they play in many important biological processes such as the transport of oxygen in blood and catalysis in mitochondria. In all of these mechanisms, porphyrins show a great affinity toward the interaction with molecules, and this property is exploited in these sensors [42]. Porphyrin-functionalized sensors have been successively used to detect cancer with respect to co-morbidities such as chronic obstructive pulmonary disease (COPD) [43] and metabolic dysfunctions [44].

Another prominent sensor technology is based on organically-capped gold nanoparticles [31]. Among the studies with these sensors, it is worth mentioning the discrimination between the first-stages and post-tumor resection cases [45]. More recently, these sensors have been applied to the detection of mutations of the epidermal growth factor receptor (EGFR) [46] and to monitor the response to anticancer treatment [47]. Other interesting devices are those based on resistive blends of polymers and carbon black [48] and colorimetric sensors [49]. Table 1 shows examples of the results achieved by electronic noses in lung cancer detection.

## 5. Conclusions

The major outcome of the combination of sensors with microelectronics is the diffusion of the analytical capabilities well beyond the traditional realms. The example of glucose sensors for diabetes is certainly paradigmatic in this regard. In the case of lung cancer, self-measurement is probably not required, but the multiplication of centers where cancer could be diagnosed early could greatly improve the management of the impact of this disease.

The hope of sensors is to simplify the acquisition of information about certain molecules known to be related to lung cancer. Biosensors are certainly unparalleled in providing exact information about markers for which the relationship with different stages of the disease is assessed and experimentally demonstrated. Biosensors are usually applied to serum, then the sample is acquired with an invasive extraction, and it is treated in order to be measured.

Besides their brilliant results, the technology of biosensors is still not mature for massive use. In particular, the loss of the properties of biomolecules when they are transferred onto a solid substrate is still a limiting factor, and biosensors are characterized by a limited lifetime. Furthermore, biosensors are disposable devices.

In the case of volatile compounds, the approach is different. The pathways describing the connection between cancer and volatile compounds are rather vague, and they may justify the occurrence of different compounds. Thus, no clear indication of which VOCs are strictly related to cancer is available. On the other hand, VOCs are attractive because the analysis is in principle non-invasive, and it can be applied at any time.

Current electronic noses have several advantages with respect to biosensors. The sensor itself is more robust, stable, and reversible, this means that it can be used for several measurements without loss of performance. Furthermore, they are ready to be produced at a large scale.

However, the lack of theoretical links between the sensor signal and the biology of cancer is a limiting factor because all the knowledge that can be obtained from these instruments is mostly phenomenological. The performance of electronic noses in a large-scale investigation including different countries and diseases has been demonstrated [51]. This study provides a solid basis to pursue the development of these sensors in order to devise a common methodology for a standardized use. This requires a strong and wide effort for the calibration of the instrument. Such effort should be aimed at eliminating all the possible interferences due to environmental aspects, the influence of co-morbidities, and the lifestyle of individuals.

## Figures and Tables

**Table 1:** Example of classification results in examples of electronic nose applications to lung cancer. The last two examples are concerned with the identification of the response to treatment; baseline is the status before treatment; partial response, stable diseases, and progressive diseases are the categories defined by the “Response Evaluation Criteria in Solid Tumors” (RECIST) approach [50].

Case	Sensitivity	Specificity	Accuracy	Reference
Lung cancer vs. control	100%	90%	93%	[43]
Lung cancer vs. control	100%	83%	90%	[44]
Lung cancer vs control	91.8%	68.8%	90.3%	[48]
Early LC vs benign nodule	75%	93.3%	86.9%	[46]
EGFR vs. wild-type	78.9%	85.3%	83.02%	[46]
Baseline vs. partial response and stable disease	93%	85%	89%	[47]
partial response and stable disease vs. progressive disease	28%	100%	92%	[47]
